# Transposable elements drive the evolution of metazoan zinc finger genes

**DOI:** 10.1101/gr.277966.123

**Published:** 2023-08

**Authors:** Jonathan N. Wells, Ni-Chen Chang, John McCormick, Caitlyn Coleman, Nathalie Ramos, Bozhou Jin, Cédric Feschotte

**Affiliations:** 1 Department of Molecular Biology and Genetics, Cornell University, Ithaca, New York 14850, USA;; 2 Department of Cell Biology, Microbiology and Molecular Biology, University of South Florida, Tampa, Florida 33620, USA;; 3 Department of Genetics and Genomic Sciences, Center for Transformative Disease Modeling, Tisch Cancer Institute, Icahn Institute for Data Science and Genomic Technology, Icahn School of Medicine at Mount Sinai, New York, New York 10029, USA

## Abstract

Cys2-His2 zinc finger genes (ZNFs) form the largest family of transcription factors in metazoans. ZNF evolution is highly dynamic and characterized by the rapid expansion and contraction of numerous subfamilies across the animal phylogeny. The forces and mechanisms underlying rapid ZNF evolution remain poorly understood, but there is growing evidence that, in tetrapods, the targeting and repression of lineage-specific transposable elements (TEs) plays a critical role in the evolution of the Krüppel-associated box ZNF (KZNF) subfamily. Currently, it is unknown whether this function and coevolutionary relationship is unique to KZNFs or is a broader feature of metazoan ZNFs. Here, we present evidence that genomic conflict with TEs has been a central driver of the diversification of ZNFs in animals. Sampling from 3221 genome assemblies, we show that the copy number of retroelements correlates with that of ZNFs across at least 750 million years of metazoan evolution. Using computational predictions, we show that ZNFs preferentially bind TEs in diverse animal species. We further investigate the largest ZNF subfamily found in cyprinid fish, which is characterized by a conserved sequence we dubbed the fish N-terminal zinc finger–associated (FiNZ) domain. Zebrafish possess approximately 700 FiNZ-ZNFs, many of which are evolving adaptively under positive selection. Like mammalian KZNFs, most zebrafish FiNZ-ZNFs are expressed at the onset of zygotic genome activation, and blocking their translation using morpholinos during early embryogenesis results in derepression of transcriptionally active TEs. Together, these data suggest that ZNF diversification has been intimately connected to TE expansion throughout animal evolution.

The Cys2-His2 zinc finger (ZNF) is a short nucleic acid binding domain widespread in eukaryotes. ZNFs are often arranged in tandemly repeated arrays, resulting in proteins with versatile nucleic acid binding ability (Wolfe et al. 2000; Klug 2010; Najafabadi et al. 2017). In metazoans, tandem-repeat ZNF genes have undergone lineage-specific expansions in gene copy number, as well as an increase in the diversity of DNA sequences that they recognize (Emerson and Thomas 2009; Najafabadi et al. 2017; Heger et al. 2020). Many ZNF proteins contain accessory domains that modulate chromatin states and transcription activity, such as the repressive Krüppel-associated box (KRAB) (Bellefroid et al. 1991). Thus, ZNFs represent the largest and most dynamically evolving transcription factor family in metazoans.

The abundance and diversity of C2H2 ZNF genes are a hallmark feature of metazoan genomes (Najafabadi et al. 2017; Heger et al. 2020), and in species whose genomes have been sequenced, ZNF gene copy number spans three orders of magnitude, from fewer than ten in many nematodes to hundreds, if not thousands, in some vertebrates, arthropods, and mollusks (Goodstadt et al. 2007; Albertin et al. 2015; Panfilio et al. 2019). Furthermore, they are frequently lineage-specific; for example, a survey of three great ape species and rhesus macaque found that ∼10% (213 out of 2253) of predicted KRAB-ZNFs (KZNFs) were species-specific, including seven from humans and 145 from orangutangs (Nowick et al. 2011). This rapid evolution contrasts with most other transcription factors, which are deeply conserved across metazoans (Degnan et al. 2009).

However, the same qualities that differentiate ZNFs from other transcription factors make them challenging to study, and consequently, there have been few attempts to produce a unified theory explaining their prevalence and recurrent expansions (Emerson and Thomas 2009; Fedotova et al. 2017). Early hints at the function of KZNFs came from work showing that deletion of the genes *TRIM28* and *SETDB1*, the H3K9 methyltransferase, led to increases in endogenous retrovirus expression in mice (Matsui et al. 2010; Rowe et al. 2010). Because TRIM28 was known to interact with the KRAB domain, this suggested that KZNFs may be involved in regulating retroelements (Thomas and Schneider 2011).

Transposable elements (TEs) are selfish genetic elements that replicate independently within their host genomes and, in most eukaryotes, comprise between 5% and 85% of the genome. Uncontrolled TE proliferation has deleterious consequences, ranging from insertional mutagenesis and genomic instability caused by ectopic recombination between TE insertions to dysregulation of gene expression stemming from the fact that many TEs carry their own promoters and regulatory sequences. Consequently, metazoans have evolved a variety of defenses to control their spread, most notably the piRNA system (Aravin et al. 2007; Czech and Hannon 2016) and the previously mentioned KZNFs (Ecco et al. 2017; Bruno et al. 2019). A hallmark of both of these defense systems is their ability to adapt to changing populations of invading TEs, which leads to selection pressure on the TEs to evade these defenses, thereby creating arms race dynamics (McLaughlin and Malik 2017).

There is growing evidence that KZNFs are engaged in such arm races with TEs (Jacobs et al. 2014; Fernandes et al. 2018; Bruno et al. 2019). KZNFs form the largest ZNF gene subfamily found in Sarcopterygii, namely, tetrapods and lobe-finned fish (Bellefroid et al. 1991; Imbeault et al. 2017). The extensive variation in KZNF copy number across species has long been appreciated (Bellefroid et al. 1991; Huntley et al. 2006), but the evolutionary forces driving this variation remained elusive until a breakthrough study showing that the number of ZNF domains in a given genome is positively correlated with retroelement copy number across a small but diverse sample of vertebrates, suggesting a coevolutionary relationship between the two (Thomas and Schneider 2011). This was bolstered by subsequent ChIP-seq experiments in humans and mice mapping the genome-wide binding of hundreds of KZNFs, which revealed that most target specific TE families (Imbeault et al. 2017; Wolf et al. 2020). Furthermore, KZNF knockouts in mice and humans lead to up-regulation of TE expression (Wolf et al. 2020; Haring et al. 2021). Mechanistic studies showed that KZNF repression of TEs is mediated via their KRAB domain, which interacts with the KAP1/TRIM28 corepressor to recruit SETDB1, among several other chromatin silencing factors (Wolf and Goff 2009; Matsui et al. 2010; Rowe et al. 2010; Ecco et al. 2017); in doing so, most KZNFs nucleate the formation of heterochromatin at their target TE loci. Together, these findings support the idea that TE proliferation drives the diversification of KZNFs.

Much less is known about the factors influencing the evolution of other ZNF families. Here, we investigate the hypothesis that interaction with TEs is a driving force underlying the molecular evolution and functional diversification of ZNF families throughout metazoans. Using a combination of comparative phylogenetics, evolutionary analyses, and in silico predictions of ZNF binding specificity, we explore the coevolutionary relationship between TEs and ZNFs in animals. We then turn our focus to a large family of ZNFs present in zebrafish, which are defined by their association with a conserved protein sequence we have termed the fish N-terminal zinc finger–associated domain (FiNZ). FiNZ-ZNF (FZNF) genes are unique to cyprinid fish and have evolved in parallel to the KZNFs found in tetrapods. Using refined gene annotations in cyprinids, we test whether these novel genes are also evolving under positive selection, consistent with ongoing arm races with TEs. Finally, by simultaneously targeting more than 400 FZNFs for knockdown during zebrafish embryogenesis, we ask whether FZNFs are capable of silencing TE expression.

## Results

### Annotation of ZNFs and TEs in metazoan genomes

**Figure 1.**
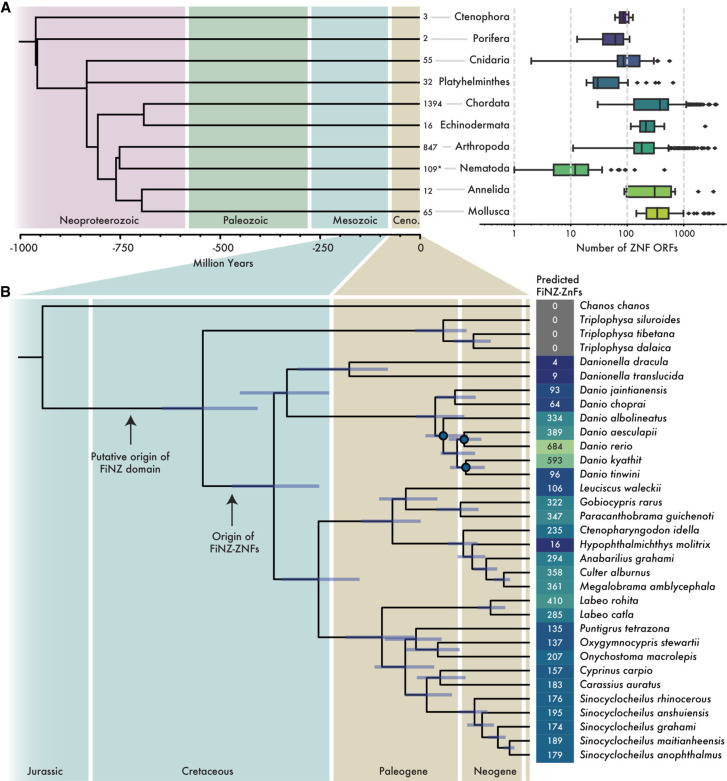
Annotation of ZNF ORFs and genes in metazoan genome assemblies. (*A*) Boxplots showing distributions of ZNF ORF counts per species in each major taxonomic phylum. *Adjacent* to boxplots are the number of representative species per phylum. (*) Number by nematodes excludes those with zero tandem repeat ZNF ORFs (defined as containing at least five repeats). Phylogeny was acquired from TimeTree5 (Kumar et al. 2022). (*B*) Maximum likelihood phylogenetic tree of Cypriniformes species with high-quality genome assemblies. Numbers represent counts of FZNF genes using improved gene annotations. Nodes marked in blue have lower than 95/95 support for Shimodaira–Hasegawa approximate likelihood ratio test/ultra-fast bootstraps, respectively.

We sought to estimate the number of ZNF genes in a large set of publicly available metazoan genome assemblies. Using open reading frames (ORFs) as a proxy for genes, we searched 3221 metazoan genome assemblies for ORFs containing ZNF domains. As many proteins contain small numbers of standalone ZNF domains (Supplemental Fig. 1), we set a threshold of at least five ZNF domains for inclusion in our data set in order to focus on those proteins with tandemly repeated ZNF arrays. This search revealed extensive variation across species, from fewer than 10 ORFs containing at least five ZNFs in most nematode worms to upward of a thousand in many vertebrates, mollusks, and arthropods (Fig. 1A; Supplemental Data 1, 2). Because estimates of ZNFs could be biased by genome assembly quality, we set a minimum scaffold N50 of 50 kb to filter out low-quality assemblies. To ensure that this threshold was appropriate, we calculated the correlation between our ZNF ORFs counts and scaffold N50 length (Supplemental Fig. 2A). This revealed a significant, but weak correlation of rho = 0.1, reassuring us that assembly quality was not unduly biasing our ORF counts.

In addition to automated counts, we performed more thorough gene annotations in cyprinid fish (Supplemental Data 2). Cyprinids, which include the widely used model organism *Danio rerio* along with many economically and culturally important carp species, are one of the most species-rich vertebrate families, with more than 1500 extant members (https://www.calacademy.org/scientists/projects/eschmeyers-catalog-of-fishes). They possess a large family of ZNF genes characterized by the presence of the FiNZ domain, a short, 28-amino-acid N-terminal sequence (Supplemental Fig. 3A). FiNZ has no significant sequence similarity to known protein domains but is highly conserved across cyprinid ZNFs and is enriched in negatively charged glutamic acid residues, a feature it shares with the KRAB domain (Bonferroni-corrected *P*-value < 0.00125 for both, relative to sequences in the SwissProt51 database) (Vacic et al. 2007). The canonical structure of FZNF genes resembles that of KZNFs, with the FiNZ domain being encoded by a single exon upstream of a second exon consisting of an array of ZNF domains (Imbeault et al. 2017).

We reannotated putative FZNF genes using a combination of BLAST (Altschul et al. 1990), HMMER (Eddy 2011), and AUGUSTUS-PPX (Keller et al. 2011; Hoff and Stanke 2018). In zebrafish, this approach yielded a total of 684 FZNF genes, a substantial increase from the number annotated in the preexisting RefSeq and Ensembl gene sets (Supplemental Fig. 3B). Almost all FZNF genes in zebrafish are located on the long arm of Chromosome 4 (Chr 4q), a region previously noted for its enrichment in TEs, ZNFs, and immunity genes, as well as a wide variety of RNA-encoding loci (Howe et al. 2013, 2016). This physical colocalization of FZNFs superficially resembles that of KZNF genes, which form large clusters of tandemly repeated genes in mammals (Tadepally et al. 2008).

Across the cyprinid family, the copy number of predicted FZNF genes varies considerably (Fig. 1B). To validate these copy number estimates, we used raw reads from four *Danio* species and compared the read coverage over FZNF genes to coverage over benchmarking universal single-copy orthologs (BUSCO) genes. In three out of four cases, there was good agreement between the two, but in the case of *Danio albolineatus*, the median read depth over FZNF genes was ∼50% greater than that of BUSCO genes, indicating that annotations provide a lower bound on copy number (Supplemental Fig. 4A). Finally, to assess whether automated ZNF ORF counts are good proxies for the number of annotated ZNF genes in a species, we compared ORF counts to those of our reannotated FZNFs, as well as independent estimates of KZNF copy number in tetrapods (Imbeault et al. 2017). In both cases, we found good agreement between the simpler ORF counts and higher-quality estimates based on gene annotations (respectively, Spearman's rho = 0.91, *P*-value = 2.6 × 10^−59^; rho = 0.97, *P*-value = 1.7 × 10^−5^) (Supplemental Fig. 4B,C).

### Correlation between ZNFs and retroelements

Early evidence for a coevolutionary relationship between TEs and ZNFs was reported by Thomas and Schneider (2011), who observed a strong correlation between ZNF and retroelement copy number in a sample of 26 vertebrate species. We sought to reproduce and expand on these results, making use of the greatly increased number of genome assemblies available today. Using the same process as for ZNF ORFs, we searched for reverse transcriptase and RNase H domains, using these as proxy for the retroelement copy number (Supplemental Data 1, 2). To address the issue of autocorrelation produced by the nonindependence of phylogenetically related species (Felsenstein 1985), we selected a single species per taxonomic family; because ZNF and TE turnover is rapid, representative species from different families have largely independent complements of both (Liu et al. 2014; Imbeault et al. 2017).

**Figure 2.**
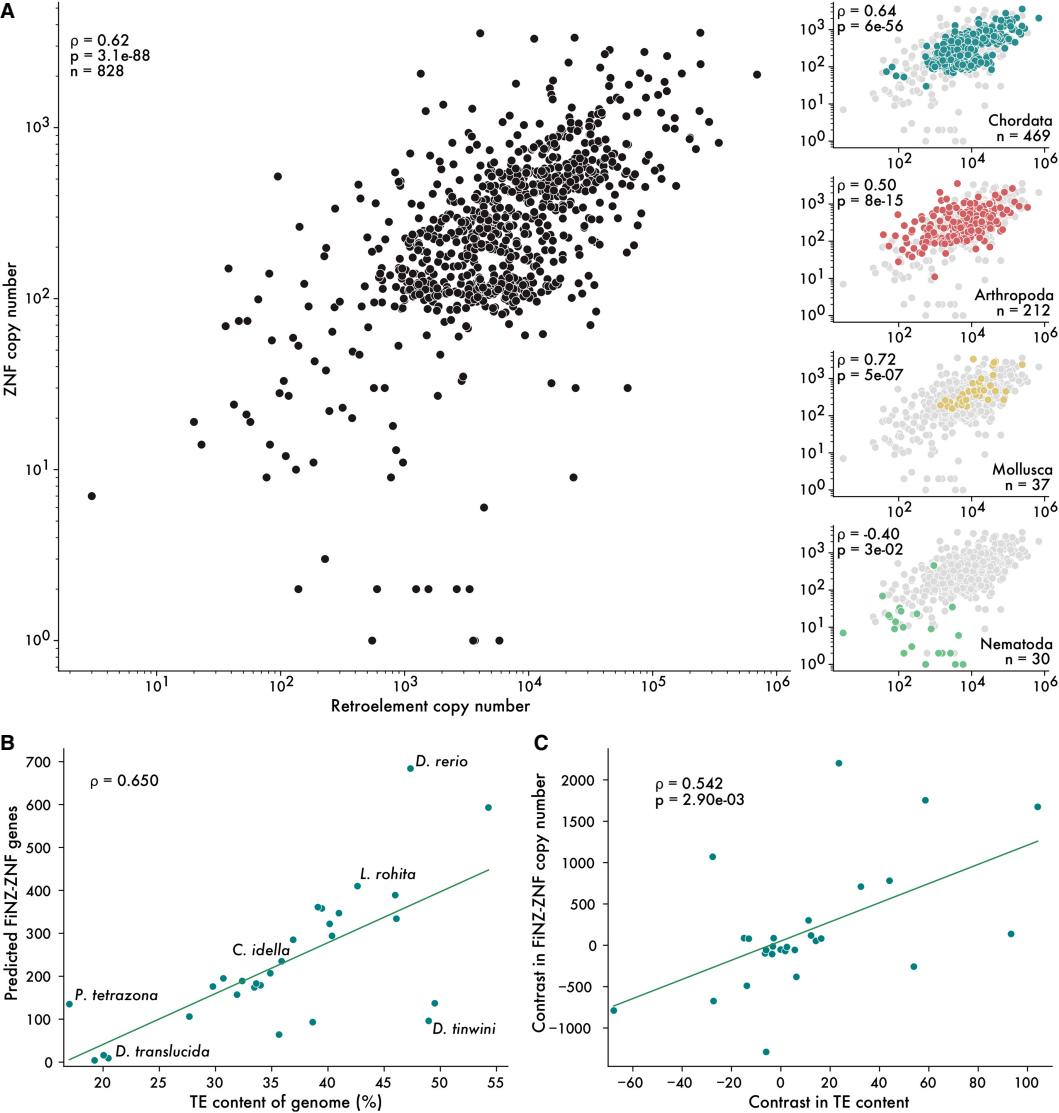
ZNF copy number correlates with genomic TE content. (*A*) Spearman's rank correlations between the estimated number of retroelement insertions and ZNF ORFs. Each point is a representative species from a taxonomic family. Subplots on the *right* show correlations for individual phyla. (*B*) Uncorrected Spearman's rank correlation between TE content and FZNF copy number in cyprinid fish. (*C*) Spearman's rank correlation on phylogenetically independent contrasts.

From the resulting set of 828 genomes, we examined the correlation between the estimated number of ZNFs and retroelement ORFs. This analysis revealed a significant positive correlation across all metazoans, as well as significant positive correlations across major phyla with at least 15 representative species, with the exception of nematode worms (Fig. 2A). Within smaller taxonomic groups, this relationship holds in most cases tested (Supplemental Data 1), with some notable exceptions. For example, birds have lost most KZNFs (Imbeault et al. 2017), such that the median number of ZNF ORFs in birds is a quarter that of other chordates (121 vs. 489); correspondingly, there is no correlation between ZNF and retroelement copy number. Similarly, we observe no correlation across dipteran flies, which possess a large ZNF subfamily known as ZAD-ZNFs; these have been studied in some depth in *D. melanogaster* and have diverse roles in heterochromatin organization but do not appear to specifically target TEs for silencing (Chung et al. 2007; Kasinathan et al. 2020; Baumgartner et al. 2022). Notwithstanding these exceptions, our analysis indicates that ZNF and TE copy numbers are positively correlated across the breadth of metazoans.

Using our refined gene annotations of FZNFs in cyprinids and improved, TE class-aware estimates of genomic TE content generated with dnaPipeTE (Goubert et al. 2015), we find that the correlation between ZNFs and TEs persists (Fig. 2B,C). Furthermore, we find that the relationship is not specific to ZNFs and retroelements but is also observed when considering DNA transposon content (Supplemental Fig. 5). These results suggest that the relationship between FZNFs and TEs is not unduly influenced by gene annotation quality and, moreover, applies to a broad range of TEs, not only transcriptionally active retroelements.

We considered two nonmutually exclusive explanations for the correlation between TEs and ZNFs. First, ZNFs may physically interact with TEs, as is the case with KZNFs (Ecco et al. 2017; Imbeault et al. 2017). Second, the TE content of the genome may have an enhancing effect on the rate of gene duplication, such that genomes with large TE content will tend to have larger gene families. The latter model is conceivable because TE accumulation is thought to affect rates of gene turnover via ectopic recombination in a copy number–dependent fashion (Charlesworth and Langley 1989; Jurka et al. 2004; Tollis and Boissinot 2012). One might predict that TE content would positively correlate not only with the copy number of ZNFs but also that of other expansive gene families. We therefore used olfactory receptors as a control case; olfactory receptors are membrane proteins known to turn over rapidly across species but are not expected to interact physically with TEs. In a sample of representative species from 103 mammalian families, we observed no significant correlation between counts of ZNF and olfactory receptor ORFs (Supplemental Fig. 6). Although this analysis does not rule out the possibility that TE content has an impact on the rate of gene duplication, it does imply that the effect is insufficient to explain the correlation observed between ZNFs and TEs.

### ZNFs are predicted to bind TE sequences

Hypothesizing that ZNF proteins interact with TEs through sequence-specific recognition of their DNA sequences, we sought to test whether ZNF binding motifs are enriched in TE sequences. Defining the binding specificity of individual ZNF genes experimentally is labor intensive, and the most successful efforts to date have involved large ChIP-based screens in human and mouse cell lines (Imbeault et al. 2017; Wolf et al. 2020). Stable cell lines are not available for most animal species, but computational methods have been developed to predict ZNF binding motifs directly from their protein sequence (Kaplan et al. 2005; Molparia et al. 2010; Persikov and Singh 2014; Najafabadi et al. 2015). Currently, these methods are not sufficient to predict binding specificity with a high degree of accuracy, making the design of reporter assays for ZNF binding impractical. However, we reasoned that if ZNFs preferentially bind TEs, then this preference should be statistically detectable when analyzing large numbers of genes. We therefore used the approach by Najafabadi et al. (2015) to predict DNA-binding motifs for all ZNF ORFs from seven metazoan species with curated libraries of TE consensus sequences: four species with a relatively large repertoire of poorly characterized ZNFs (octopus, zebrafish, rice weevil, and sea urchin), two positive control species known to use ZNFs to target TEs (human and mouse), and an expected negative control species, *D. melanogaster*, whose ZNFs are apparently not targeting TEs directly (Supplemental Data 3).

**Figure 3.**
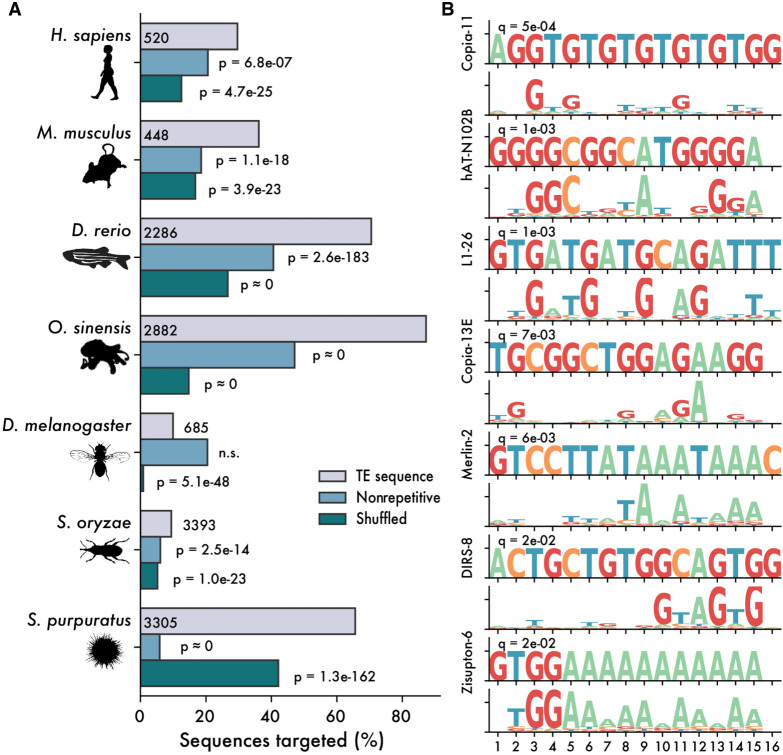
ZNFs are predicted to bind TE sequences. (*A*) Percentage of sequences matched by predicted ZNF motifs, with a *Q*-value cutoff of 0.05. Gray bars represent matches to libraries of consensus TE sequences, and blue bars represent matches in equally sized libraries of nonrepetitive genomic sequence. Green bars represent shuffled motifs, searched against consensus TE libraries. *P*-values are calculated as one-tailed binomial tests for the probability of observing at least x matches in the TE library, using binding frequency to nonrepetitive sequences or with shuffled motifs as a base probability. (*B*) Selected matches between *Danio rerio* consensus TE sequences and ZNF ORFs.

First, we used previously published ChIP-exo data for human KZNFs (Imbeault et al. 2017) to confirm that computationally predicted DNA-binding motifs were similar to those experimentally determined. Searching for each of 236 experimentally determined KZNF binding sites in the set of predicted binding motifs, we found that 11 (5%) produced significant matches to their predicted counterpart (*Q*-value < 0.05), compared with zero matches after shuffling the predicted motifs. Similar results have been shown by others working with mice (Wolf et al. 2020). As a second positive control, we compared the predicted binding motifs for human and mouse ZNF ORFs to libraries of their consensus TE sequences and observed a significant enrichment of predicted matches within TEs, compared with matches using shuffled motifs (Fig. 3A). This result is expected because KZNFs are known to target TEs and confirms that predicted binding sites recapitulate, at least in part, experimentally obtained motifs. For example, we were able to recapitulate human ZNF320 binding to LTR14a and ZNF483 binding to L1PA7 (Supplemental Fig. 7). These controls show that although computational prediction of ZNF binding specificity remains challenging, it is sufficient to capture a proportion of biologically relevant binding activity.

We therefore turned our attention to the four species whose ZNFs are less studied (zebrafish, octopus, rice weevil, sea urchin). For each species, we predicted binding motifs for all ZNF ORFs and then examined the frequency of these motifs in corresponding libraries of consensus TE sequences (Fig. 3A). Using a false-discovery threshold of q = 0.05, we searched for significant matches between our predicted ZNF binding motifs and TE consensus sequence and then compared them to equivalently sized libraries of nonrepetitive genomic sequence. For all four species, we observed a significant enrichment of matches to the TE sequences compared with nonrepetitive sequences (Fig. 3A). In contrast, we do not observe such enrichment for *D. melanogaster*. Rather, for that species, we found a greater percentage of nonrepetitive sequences matched by predicted ZNF binding motifs (Fig. 3A). This finding is consistent with previous functional studies of *Drosophila* ZAD-ZNFs (Kasinathan et al. 2020) and our observation that the copy number of dipteran ZNFs does not correlate with the number of TE copies (Supplemental Data 1). Notably, we also found that in all species both retroelements and DNA transposons were predicted to be targeted by ZNFs (Supplemental Data 3).

As an additional control, we repeated the analysis using shuffled motifs and found in all species that the predicted motifs bound a higher percentage of TE sequences compared with the shuffled motifs (Fig. 3A). We note, however, that shuffled motifs still recognize a substantial fraction of sequences in most species, likely because ZNFs are often predicted to bind simple sequence repeats or homopolymeric tracts that would be unaffected by shuffling (e.g., Fig. 3B). Finally, to test species specificity, we searched the *D. rerio* TE library using human ZNFs and compared these to zebrafish ZNFs. We found that zebrafish ZNFs were far more likely to target zebrafish TEs than were human ZNFs (odds ratio = 29.5, *P* ≈ 0.0, Fisher's exact test). These results suggest that the preferred binding of ZNFs to TE sequences is not unique to mammalian KZNFs but is likely a general property of many metazoan ZNF families. However, direct experimental validation of ZNF binding in non-mammalian organisms will be required before we can be confident that these predictions reflect reality.

### FZNFs are evolving under positive selection in zebrafish

TEs evolve rapidly and are under strong selection to escape repression by their hosts; many KZNF proteins are in direct competition with TE families, leading to signatures of arm races between the two (Jacobs et al. 2014; Fernandes et al. 2018; Bruno et al. 2019). One such signature is positive selection (i.e., adaptive evolution) acting on the DNA contacting residues of ZNFs, namely, those involved in TE recognition (Emerson and Thomas 2009). We therefore tested for evidence of positive selection in *D. rerio* FZNFs by comparing rates of synonymous and nonsynonymous substitution (*d*_N_/*d*_S_).

We restricted our analyses to seven clades of recently duplicated paralogs unique to *D. rerio* with a minimum of 10 members (for details, see Methods). Using PAML (Yang 2007), we performed likelihood ratio tests to compare two models of evolution for each clade: one in which the genes were evolving under purifying selection, and one in which some sites were assumed to be under positive selection (M2 vs. M2a). In four of these seven clades, the model featuring positive selection (M2a) was significantly favored (*P*-value < 0.01, likelihood ratio tests) (Supplemental Data 4). Furthermore, in two clades we found that base-contacting residues of ZNF domains were significantly enriched for values of *d*_N_/*d*_S_ > 1 (odds ratio > 2.45, *P*-value ≤ 0.05; Fisher's exact test). As an alternative to *d*_N_/*d*_S_ analyses, which are dependent on accurate gene alignments, we calculated the sequence entropy at positions in the canonical ZNF domain for each species in Figure 3. This revealed that base-contacting residues are among the most variable in sequence, with the possible exception of *D. melanogaster* (Supplemental Fig. 8). These data, combined with the enrichment of ZNF predicted binding sites in TE sequences, suggest that zebrafish ZNFs, and likely those of many other species, are evolving adaptively to target TEs.

### FZNFs are expressed in distinct waves during embryogenesis

TEs—retroelements especially—are highly active during metazoan embryogenesis, as this provides them with the opportunity to transpose in the cells giving rise to the adult germline, thus ensuring the vertical inheritance of new insertions (Haig 2016; Rodriguez-Terrones and Torres-Padilla 2018; Ansaloni et al. 2019; Chang et al. 2022). Previous work has shown that zebrafish ZNFs are expressed at the onset of zygotic genome activation (ZGA), mirroring the pattern of KZNFs in human and mice and consistent with a potential role in TE silencing (White et al. 2017; Pontis et al. 2019; Hadzhiev et al. 2023). We sought to further explore the timing of FZNF expression, predicting that if they are deployed to repress TEs, their expression should overlap with that of TEs. Using our de novo FZNF annotations, we remapped previously published RNA sequencing data covering the first 24 h of zebrafish development (White et al. 2017). Setting a lower limit of 0.5 transcripts per million (TPM) to call genes as expressed, we find that approximately half of all FZNFs (306 out of 684) are expressed during early development.

**Figure 4.**
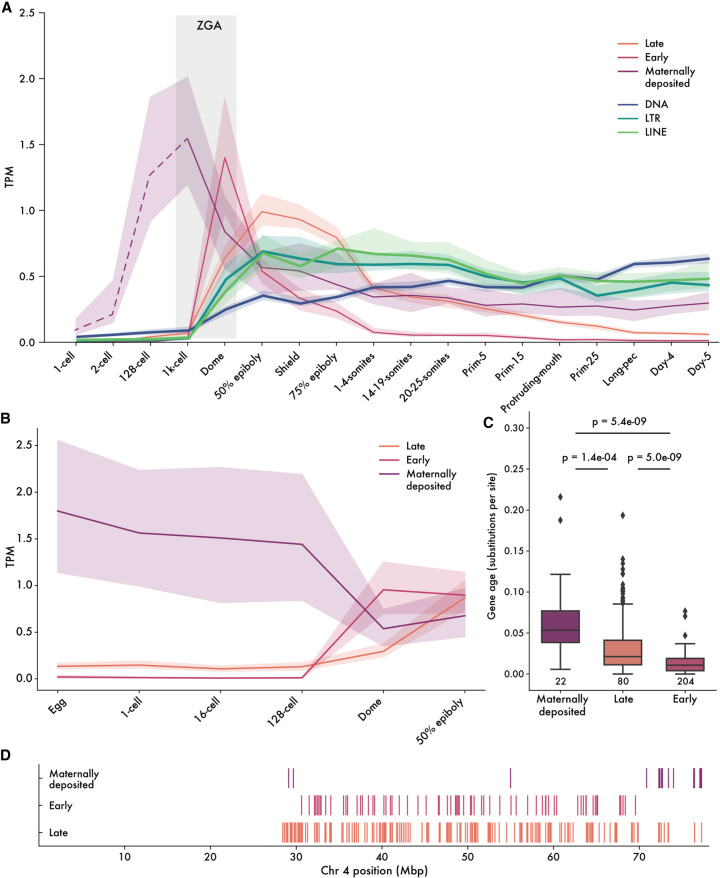
Embryonic expression of FZNF genes in zebrafish. (*A*) Median expression trajectory of 306 genes with expression of at least 0.5 TPM in at least one stage, separated by cluster. Shaded borders represent 90% confidence intervals. Light gray bar shows approximate timing of ZGA. Expression trajectory of maternally deposited FZNFs before the 1000-cell stage is dashed to reflect the fact that poly(A)-selected RNA sequencing is not an accurate reflection of true mRNA levels before ZGA. (*B*) FZNF expression from rRNA-depleted reads, before ZGA. (*C*) Maternally deposited genes are significantly older, whereas early-stage FZNFs are young and generally unique to *D. rerio*. Ages were quantified as branch lengths on a neighbor-joining phylogenetic tree generated from pairwise distances. (*D*) The majority of maternally deposited FiNZ genes are located in a specific, subtelomeric cluster. Each vertical line represents the midpoint of an expressed FZNF gene.

Recently published work identified two distinct waves of ZNF expression in zebrafish, named “sharp peak” and “broad peak” (Hadzhiev et al. 2019, 2023). Sharp peak FZNFs share a distinct promoter architecture consisting of a clearly identifiable TATA-box and are expressed in the minor wave of ZGA, followed shortly after by TATA-less broad peak ZNFs in the major wave. With our updated FZNF annotations, we recapitulated these findings, observing the expression of 80 sharp peak and 204 broad peak genes, hereafter termed “early” and “late” based on the timing of their expression (Fig. 4A). The timing of these peaks overlaps with that of most TE families and, in the case of early-stage FZNFs, precedes almost all zygotic TE transcription.

We also found a small subset of approximately 22 FZNF genes with maternally deposited transcripts (Fig. 4A). To our knowledge, these have not previously been described, likely owing to their incomplete annotation in Ensembl and RefSeq gene sets. Furthermore, maternally deposited mRNAs in zebrafish and other animals are, initially, only partially polyadenylated (Potireddy et al. 2006; Cui et al. 2013; Winata et al. 2018) and are therefore difficult to detect when using poly(A)-selected RNA-seq platforms. As the White et al. (2017) data falls into this category, we remapped rRNA-depleted reads from a second data set covering early embryogenesis (Winata et al. 2018), which confirmed that these maternally deposited transcripts are indeed present in both the egg and one-cell zygote (Fig. 4B).

Given the markedly different expression pattern of maternally deposited FZNFs, we looked for other features differentiating them from their zygotically expressed counterparts. Although the majority of *D. rerio* FZNFs are recently duplicated, particularly early-stage FZNFs, those that are maternally deposited are conserved across species and are significantly older (Fig. 4C; Supplemental Data 5). Moreover, they are physically colocalized in the subtelomeric region of Chr 4q (Fig. 4D), which lacks the repeat density that characterizes much of Chr 4q (Chang et al. 2022). The age of maternally deposited genes, as well as their degree of species conservation, is hard to reconcile with a role in TE targeting, as TEs turn over rapidly and are frequently species-specific. Rather, the deeper conservation and physical clustering of these maternally deposited FZNFs suggests a role in development that differs from that of early- and late-stage FZNFs.

### FZNFs repress LTR retroelement expression during ZGA

**Figure 5.**
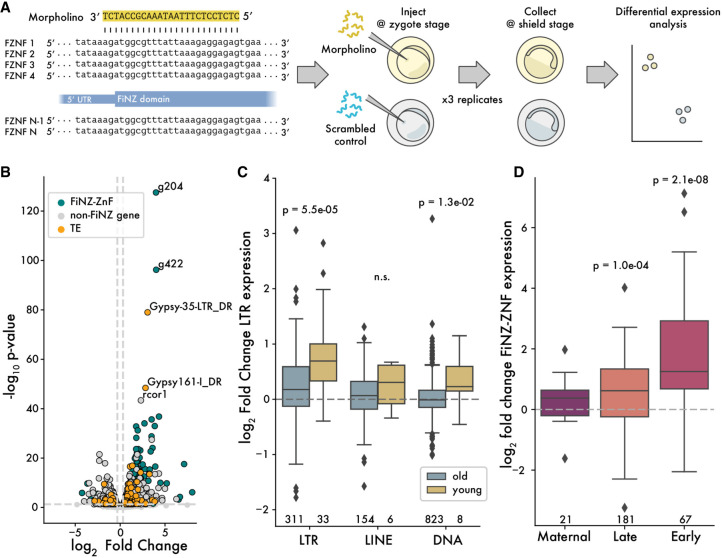
Up-regulation of LTR retroelements in response to FZNF knockdown. (*A*) Cartoon showing design of MO-based FZNF knockdown experiment. (*B*) Log_2_ fold change versus *P*-value, comparing FiNZ translation blocking MO to scrambled MO control. (*C*) Log_2_ fold change comparison between old and young TE families, with “young” being defined as those whose median insertion age was less than 0.01 substitutions per site. *P*-values are Wilcoxon rank-sum tests comparing old and young in each TE class. (*D*) Log_2_ fold change of different FZNF categories. *P*-values are binomial tests for each category, assessing likelihood of seeing at least x up-regulated genes, assuming a null hypothesis in which there is a 50% chance of being either up- or down-regulated. Numbers *below* boxes in *C* and *D* give the number of data points per box.

Next, we turned to the function of FZNF genes. Based on their embryonic expression, predicted binding specificity, and strong correlation with TE content, we predicted that FNZF genes play a role in TE silencing. If true, then knocking down their expression ought to lead to increased TE expression. To test this, we used a Morpholino oligonucleotide (MO) strategy aimed at blocking translation of most FZNF proteins simultaneously. By aligning the first exon of the FZNF annotations from zebrafish, we were able to design an MO predicted to target the translation start site of 447 out of 684 annotated FZNFs (Fig. 5A). We injected zebrafish embryos with either this MO or a scrambled control and then compared gene and TE expression using RNA-seq. Embryos were collected for sequencing at shield stage, as this corresponds with a period at which many TE families and most FZNFs are robustly expressed (Fig. 4A).

After sequencing and read mapping, we performed quality controls before comparing TE expression between treatment and control groups. First, we performed principal component analyses of batch-corrected samples, which showed clear separation between the FZNF knockdown and control groups (Supplemental Fig. 9A). Using a false-discovery rate threshold of 0.05 and an absolute log_2_ fold change of at least 0.32 to call differentially expressed genes and TE families, we observed a total of 999 differentially expressed genes, excluding FZNFs and TEs (Supplemental Data 6). Among these, a Gene Ontology enrichment analysis revealed no notable enrichment for biological processes beyond general terms related to embryonic development, metabolic processes, and translation (Supplemental Data 7). Importantly, we did not observe any enrichment for stress response pathways, nor did we observe a significant change in *tp53* levels (log_2_ fold change = −0.01), which can cause off-target effects in MO knockdowns (Bedell et al. 2011).

As the expression of TEs and FZNF genes is highly dynamic during the first 24 h of zebrafish development (Laue et al. 2019; Guo et al. 2021; Chang et al. 2022), we sought to ensure that the embryos were staged appropriately, as differences in the relative stage of FZNF knockdown and control groups could lead to artifactual observations of differential expression. We therefore compared our samples with samples from White et al. (2017), covering 50% epiboly, shield stage, and 75% epiboly. Treating these samples as additional, batch-corrected controls, we found that our samples clustered with shield stage samples from White et al. (2017), indicating that our staging is accurate (Supplemental Fig. 9B,C).

To assess whether FZNFs are involved in TE silencing, we asked whether TE expression was up-regulated in the FZNF knockdown group. Looking at the overall number of differentially expressed TE families, we found that TEs were significantly more likely to be up-regulated than down-regulated, consistent with derepression following FZNF knockdown (odds ratio = 3.78, *P*-value = 6.1 × 10^−8^, Fisher's exact test) (Fig. 5B; Supplemental Fig. 9D,E). Separating by TE class, we found that this trend was driven predominantly by LTR elements (odds ratio = 6.56, *P*-value = 1.2 × 10^−8^, Fisher's exact test) (Supplemental Fig. 10A). Because LTRs include many of the youngest TE families in the zebrafish genome (Chang et al. 2022), we reasoned that their enrichment among up-regulated genes and TE families may reflect a tendency for young, active families to be most intensively targeted by FZNFs. We therefore compared young and old TE families to each other, defining the former as those whose insertions averaged fewer than 0.01 substitutions per site (Chang et al. 2022). This analysis revealed that the magnitude of derepression following FiNZ-knockdown was significantly greater among young families (*P*-value = 5.5 × 10^−5^, Wilcoxon rank-sum test) and that this trend held across different TE classes (Fig. 5C). Thus, FZNF knockdown leads to significant derepression of young, potentially active TE families.

In addition to age, we also looked at the timing of expression, because by collecting at “shield” stage (∼6 h post fertilization), we would not be able to observe the effects on those TEs whose expression peaked at later time points. Using hierarchical clustering to separate TEs into groups with expression peaks either before or after shield stage, we found that those TE families that were expressed earlier were significantly more up-regulated than those expressed later (Supplemental Fig. 10B).

The genes most up-regulated by FZNF knockdown were FZNFs themselves, for example, g204 and g422 (Fig. 5D). Although we cannot currently rule out the possibility that MOs inhibit the degradation of their target sequences, to our knowledge, such effects have not previously been reported in the literature. Alternatively, these results suggest that FZNF genes are themselves targeted for repression by FZNF proteins, thus forming a negative feedback loop. In this way, their expression would be robustly switched off once sufficient FZNF protein had been generated, without the need for additional regulatory control. Such negative feedback loops have recently been documented for mammalian KZNFs (Pontis et al. 2022).

## Discussion

In this work, we observed a deep coevolutionary relationship between TEs and ZNFs that spans the breadth of metazoans. This is most clearly seen in the strong positive correlation between TE and ZNF copy numbers, which persists across at least 750 million years of animal evolution and is independent of the accessory domains that characterize different tandem repeat ZNF families. We also found that ZNFs from a range of metazoans are predicted to bind TE sequences and, in the case of FZNFs in zebrafish, are evolving under positive selection acting on the DNA-binding ZNF domains. Finally, through simultaneous knockdown of a large subset of FZNFs during zebrafish embryogenesis, we showed that this family represses young, recently active TEs.

The extensively duplicated ZNF families of metazoans are rarely observed in other eukaryotic taxa, where instead, small-RNAs acting in concert with Argonaute proteins are the primary drivers of TE heterochromatinization (Hall et al. 2002; Volpe et al. 2002; Najafabadi et al. 2017; Allshire and Madhani 2018; Heger et al. 2020). Thus, ZNF-based TE targeting is likely to be a metazoan innovation. However, there are several metazoan groups whose ZNF families appear to behave atypically. For example, we found no positive correlations between ZNFs and retroelements in dipteran flies or nematode worms, nor did we observe a binding preference for TEs in *Drosophila* ZNFs. However, in *Drosophila* the piRNA system (itself a branch of the Argonaute-siRNA system) is directly involved in pretranscriptional silencing of TEs during embryogenesis*,* with *piwi* knockdowns resulting in a failure of H3K9me3 to establish over otherwise silenced TE loci (Sienski et al. 2012; Fabry et al. 2021; Wei et al. 2021). Similarly, in nematode worms, which typically have few tandem-repeat C2H2 ZNF genes, Argonaute genes have been extensively duplicated (Buck and Blaxter 2013). Although research into the function of these Argonaute homologs remains in the early stages (Seroussi et al. 2023), both the dipteran and nematode cases point to potential functional overlap between the Argonaute/piRNA system and ZNFs.

It is still an open question whether silencing of TE transcription per se, is the primary function of metazoan ZNFs. In addition to their TE silencing activities, KZNFs frequently target non-TE sequences, such as satellite repeats, gene promoters, and even other KZNFs (Imbeault et al. 2017). There are also examples in both mouse and human of conserved clusters of KZNFs that target TEs younger than themselves, implying they had targets predating the emergence of their current TE families (Wolf et al. 2015; 2020; Imbeault et al. 2017). These observations have led others to propose that, rather than simply repressing TEs, KZNFs play an important role in their domestication by taming their *cis*-regulatory activities and facilitating their integration into existing gene regulatory networks (Ecco et al. 2017; Bruno et al. 2019; Pontis et al. 2019). Under this model, KZNFs act as tolerogenic agents promoting the evolution of species-specific gene regulatory networks. Although likely true, this feature cannot be the initial driver of their rapid evolution, because TE domestication is a process that takes place over many generations, whereas selection primarily acts on currently extant individuals (Sniegowski and Murphy 2006).

Although depletion of both KZNFs and FZNFs unleash TE expression, there are several paradoxical observations that challenge the idea of ZNF as an adaptive defense system against TE invasion. First, ZNFs evolve slowly relative to the speed at which novel TE families can establish and propagate within a species. For example, the DNA transposon P-element spread throughout all wild populations of *D. melanogaster* in the space of ∼30 yr, and the retrovirus KoRV has been spreading in koalas exogenously and endogenously over a similar period (Anxolabéhère et al. 1985; Tarlinton et al. 2006). In contrast, observations in primates imply that the emergence of KZNF repressors takes place over millions of years (Ecco et al. 2017; Imbeault et al. 2017). In contrast, the piRNA system is able to adapt almost immediately to new TEs, as TE insertions themselves form the raw material for piRNAs—indeed, in the case of both KoRV and P-element, piRNAs have already emerged to mitigate their deleterious effects (Brennecke et al. 2008; Yu et al. 2019). Thus, with respect to their ability to quickly respond to novel TEs, the piRNA system appears better suited as a front-line defense than ZNFs.

We therefore propose an alternative to transcriptional repressor hypothesis, in which metazoan ZNFs instead serve as genome stabilizers, protecting DNA from ectopic recombination by nucleating heterochromatin to repetitive regions of the genome. Although heterochromatin is primarily associated with gene silencing, it also has a strong suppressive effect on homologous recombination and is essential for maintaining genome stability (for reviews, see Grewal 2010; Sasaki et al. 2010; Kent et al. 2017; Allshire and Madhani 2018). TE-mediated chromosomal rearrangements are highly deleterious and associated with several hereditary diseases (Callinan and Batzer 2006), both in germline and somatic tissues, as evidenced by the large number of human cancers associated with TE-induced structural variants (Anwar et al. 2017). Accordingly, the propensity of TEs to cause deleterious chromosomal rearrangements is thought to be a critical factor preventing their accumulation in genomes, particularly because the theoretical probability of recombination between TE insertions increases quadratically with copy number (Charlesworth and Charlesworth 1983; Langley et al. 1988). This hypothesis—that selection against ectopic recombination is critical to preventing TE accumulation—has been supported by observations from both empirical and simulated data (Petrov 2003; Song and Boissinot 2007; Dolgin and Charlesworth 2008; Kofler et al. 2012), although others have drawn different conclusions (Duret et al. 2000; Wright et al. 2003).

Thus, the use of sequence-specific ZNFs to deposit heterochromatin over discrete TE families might explain in part why some organisms can sustain large-scale amplification of TE families, as seen in octopuses, weevils, sea urchins, cyprinid fish, and mammals, to name a few highlighted in this study. Moreover, both KZNFs and FZNFs, along with other highly duplicated gene families, are also found in regions of the genome marked by H3K9me3, further suggesting that stabilization of repeats, rather than suppression of TE transcription, is the purpose of heterochromatin deposition at these locations (Hawkins et al. 2010; Kundaje et al. 2015). If genome stabilization is the principal function of most metazoan ZNFs, then it relieves the requirement for rapid adaptation to novel TEs, because the effect of TEs on rates of nonallelic homologous recombination are not felt until the TEs in question have reached significant copy number. Similarly, it would explain why satellite sequences or nonautonomous DNA elements, which do not need to be transcribed to amplify, appear to be targets of many ZNFs.

Moving forward, the FZNF family will be an important model for understanding the function and evolution of metazoan ZNFs, and many questions raised in this study remain unanswered. For example, what is the mechanism by which FZNFs exert their repressive effect? Although zebrafish FZNFs are, in aggregate, predicted to target TE sequences (Fig. 3), we have not yet validated the binding of individual ZNF-TE pairs, and it therefore remains possible that they achieve silencing via a mechanism other than DNA-binding. Similarly, we do not yet know whether the FiNZ domain itself is capable of recruiting the protein complexes required for heterochromatin formation. Although KRAB and FiNZ have similar sequence composition and secondary structure, the KRAB domain recruits the H3K9me3 writer SETDB1 through an intermediary cofactor, TRIM28, which is absent in zebrafish. However, two close relatives of TRIM28, namely, TRIM24 and TRIM33, are present in zebrafish and have been shown to silence endogenous retroviruses in mice (Boudinot et al. 2011; Margalit et al. 2020). Furthermore, structural predictions suggest that TRIM33 physically interacts with distantly related “KRAB-like” ZNF sequences present in fish (Carotti et al. 2022), suggesting that it may be the cofactor of FiNZ.

Much remains to be discovered about the role of ZNF genes in metazoan evolution, but with the wealth of high-quality genome assemblies and improved tools for manipulating gene expression in a variety of organisms, it is now possible to answer many of the long-standing questions about this extraordinary gene family. In this work, we have shown that the coevolutionary relationship between TEs and ZNFs extends well beyond KZNFs and is likely a metazoan innovation for TE silencing and/or stabilization. In zebrafish, FZNFs have independently expanded to repress young, recently active TE families during early embryonic development, and across animals, there are dozens of other such uncharacterized ZNF families. Many of these families are likely to reveal exciting new biology and may have potential uses in the development of genome engineering tools.

## Methods

### Predicting ZNF ORF copy number

Initial estimates of ZNF ORF copy number were achieved by extracting all possible ORFs from 3221 representative metazoan genomes available from the NCBI Assembly database (https://www.ncbi.nlm.nih.gov/assembly), as of December 6, 2021. To facilitate this process, we set minimum and maximum ORF sizes of 375 and 10,000 bp, respectively. The resulting amino acid sequences were searched for potential C2H2 ZNF domains using the hmmsearch command line tool from the HMMER suite (Eddy 2011) and the Pfam “zf_C2H2” (Pfam: PF00096) HMM as a query profile (Mistry et al. 2021). Resulting hits were merged if they were within 100 residues of each other and were filtered to exclude sequences with fewer than five ZNF domains in total.

### Annotation of FZNF genes

For improved annotations of FZNF genes in 32 cypriniform fish and a milkfish outgroup, we first identified genomic regions containing candidate FZNF genes using BLAST to search for matches with a consensus FiNZ domain sequence. This consensus sequence was generated from a set of *D. rerio* FZNF genes previously identified as being expressed during development (White et al. 2017). Using the “PPX” module of AUGUSTUS v3.3 (Keller et al. 2011; Hoff and Stanke 2018), we performed a first pass of the genomic regions identified in our set of cypriniform species, using a protein profile generated from the previously mentioned set of *D. rerio* annotations. To avoid biasing our search toward zebrafish, we used the results of this round of annotation to generate a new FZNF profile generated by sampling genes from all species. We repeated the above procedure once and performed a final filtering step with HMMER (Eddy 2011) to identify annotated genes containing both the FiNZ domain and ZNF domain (PFAM: PF00096).

For *D. rerio* specifically, we produced a separate, high-quality set of gene predictions by retraining AUGUSTUS specifically for FZNFs using a manually curated set of Ensembl predictions. This allowed us to generate predictions for 5′ and 3′ untranslated regions, for use downstream in analyses of gene expression. We explored the effect of including transcripts generated by Trinity (Grabherr et al. 2011) as hints for AUGUSTUS but found that these reduced the quality of resulting annotations, likely as a result of the difficulty of assembling accurate transcripts for such repetitive genes. Full parameter details are available online.

### Predicting genomic TE content

We used two approaches to estimate genomic TE content. In the first, we focused on retroelements, using an identical approach to that which we used to estimate ZNF ORF number. In place of the Pfam zf_C2H2 domain, we instead used a selection of reverse transcriptase and RNase H profiles (PF00078, PF07727, PF13456, PF00075, PF17917, PF17919). Resulting hits were merged as performed for ZNFs, but no restrictions were placed on the number of hits per ORF. For more careful estimates of TE content for use when focusing on FZNF genes, we estimated the TE-derived proportion of genomes using dnaPipeTE (Goubert et al. 2015), a tool that offers considerable speed improvements over RepeatMasker/Modeller with comparable accuracy. This program requires short reads and genome size as input: For the former, we simulated reads using ART (Huang et al. 2012), and for the latter, we used genome assembly size as a proxy for true genome size.

### Cypriniformes phylogeny

To generate a phylogenetic tree of the Cypriniformes order (required to account for phylogenetic nonindependence in comparative genomic analyses), we first used the Actinopterygii-specific BUSCO database to extract intact single-copy orthologues from our set of 33 species. We then selected those protein sequences found in at least 10 out those species, producing a final set of 3581 proteins. These were aligned separately using MAFFT v7.475 with the parameters –globalpair and –maxiterate 1000 (i.e., ginsi) (Katoh and Standley 2013) and trimmed to remove large insertions or poorly aligned regions using trimAl v1.4.rev15 with the –automated1 parameter set (Capella-Gutiérrez et al. 2009). All resulting files were combined to produce a concatenated supergene alignment, which was used as input when generating a time-calibrated phylogeny.

IQ-TREE v2.0.6 was used to generate a maximum likelihood tree from a partitioned analysis of the supergene alignment (Chernomor et al. 2016; Hoang et al. 2018; Minh et al. 2020). Ultrafast bootstraps and Shimodaira–Hasegawa approximate likelihood ratio tests were used to determine branch support values, with 5000 and 1000 replicates, respectively. A time-calibrated tree was generated using the integrated LSD2 module (To et al. 2016), constraining the date of the split between Gonorynchiformes and Cypriniformes (i.e., between *Chanos chanos* and all other species) to 162 million years ago, based on a recent comprehensive phylogeny of teleost fish (Hughes et al. 2018).

### Predicting ZNF binding sites

To predict ZNF binding sites, we made use of the previously published tool, ZiFRC, with ZNF ORFs identified from our initial genome searches as input (Najafabadi et al. 2015; 2017). To test for enrichment of predicted ZNF binding sites in various sequence sets, we used FIMO and Tomtom from the MEME suite of motif enrichment tools (v5.3.3) (Bailey et al. 2015). First, Tomtom was used to compare our predicted motifs to motifs obtained from human ChIP-exo data sets (Imbeault et al. 2017). Following this positive control, we used FIMO to calculate motif enrichment in three separate cases for each species.

In the first case, we searched our library of predicted ZNF binding sites against libraries of TE consensus sequences. For human, mouse, fruit fly, and zebrafish, these libraries were taken directly from Repbase v26.10; in the case of sea urchin and rice weevil, they were provided by direct communication with the investigators of previously published studies on the TE content of these species (Panyushev et al. 2021; Parisot et al. 2021); and, in the case of octopus, we generated a TE consensus sequence library using RepeatModeler v2.0.1 (Flynn et al. 2019).

For the second case, we searched a library of randomly selected nonrepetitive genomic sequences, selected using BEDTools v2.30.0 (Quinlan and Hall 2010); this was performed in a manner ensuring that, for each species, the resulting library size and distribution of sequence lengths were identical to those of the TE sequence library. In the third case, we searched the TE consensus sequences again, this time using shuffled binding motifs. Finally, we performed a cross-species comparison between zebrafish and human using FIMO.

### Testing for positive selection

To test for evidence of positive selection in FZNF genes, we first searched for recently duplicated in-paralogs. As multiple sequence alignments of ZNF genes can be unreliable, we calculated pairwise Needleman–Wunsch alignments between all pairs of 6732 annotated genes from 29 cypriniform species with at least one FZNF gene, using the “needle” command line tool from the EMBOSS suite, v6.6.0 (Rice et al. 2000). The resulting pairwise distance matrix was used as input to generate a neighbor-joining phylogenetic tree, using the EMBOSS “fneighbor” tool. This gene tree was used to identify clades of recently duplicated in-paralogs within the cypriniform species tree. We selected seven clades from *D. rerio*, in which each clade contained at least 10 members, and all members were within 30 amino acids of each other in length. Each one of these clades was realigned with Prank, using a codon substitution matrix (Kosiol et al. 2007; Löytynoja 2014), and all gaps in the resulting alignment were removed using trimAl v1.4.rev15 (Capella-Gutiérrez et al. 2009). Finally, guide trees were generated for each clade using IQ-TREE v2.0.6 with a codon substitution model (Minh et al. 2020).

To test for evidence of positive selection, we used the “codeml” tool from the PAML4 software suite (Yang 2007). We compared two sets of nested site models: first, M1a (NearlyNeutral) and M2a (PositiveSelection), and second, M7 (beta) and M8 (beta & ω). The transition-to-transversion ratio, κ, was calculated from the guide tree, whereas the *d*_N_/*d*_S_ rate ratio, ω (i.e., the parameter being tested), was estimated from the data. Likelihood ratio tests with two degrees of freedom were used to compare M1a to M2a, and M7 to M8, respectively.

### Reanalysis of zebrafish developmental transcriptome data

To assess FZNF gene and TE expression, we first used STAR (v2.7.5a) (Dobin et al. 2013) in multimapping mode to realign reads from White et al. (2017) to the *D. rerio* GRCz11 reference genome, setting the maximum intron/read pair gap size at 500,000. To count reads from genes and TEs simultaneously, we used TEtranscripts-2.2.1's TEcount tool in multimapper mode, with a GTF file combining Ensembl gene annotations with our FZNF annotations, along with TE locations as provided by Chang et al. (2022). We used GTFtools v0.8.0 (Li et al. 2022) to calculate the median length of each gene and used these measurements to calculate the TPM for each gene/TE, allowing comparison of expression across stages. Specifically, for each transcript we calculate the sum of the union of exons. For genes, we then take the median length of transcripts associated with each gene, whereas for TEs we use the sum of all insertions in the genome, thus partially controlling for insertion copy number.

### Morpholino knockdown of FZNF translation

We ordered a translation blocking MO with the sequence “CTCTCCTCTTTAATAAACGCCATCT” from Gene Tools, which was sufficient to target 447 of our predicted FZNF mRNA transcripts with no mismatches. Wild-type, Tübingen strain zebrafish were maintained on a 14-/10-h light–dark schedule at 28°C. Individual breeding pairs of male and female fish were separated overnight to induce naturally synchronized spawning and fertilization the following morning. To knockdown FZNF expression, we injected 4.5 ng of the custom MO into fertilized *D. rerio* zygotes, and in parallel, we injected embryos with the same quantity of a randomized control, consisting of a mixture of up to 4^25^ possible sequences. Embryos were collected at shield stage and stored at −20°C immediately after collection. Each pair of injections was repeated in triplicate, such that 23 treatment and 19 control embryos were in the first batch, 30 treatment and 30 control in the second, and 31 and 30, respectively, in the third. Total RNA extraction was performed using the Qiagen RNeasy kit.

### RNA sequencing

We outsourced cDNA library preparation and sequencing to Novogene. Samples were shipped in dry ice, and after quality controls, strand-specific libraries were prepared using poly(A) enrichment. These libraries were pooled and sequenced in 150-bp paired-end mode using the NovaSeq 6000 platform. After receiving sequencing data, we performed quality assessment using FastQC (v0.11.9) (https://www.bioinformatics.babraham.ac.uk/projects/fastqc/) and then mapped and counted reads according to the protocol previously described for reanalysis of White et al. (2017) data. Following this, differential expression analyses were performed using the DESeq2 R package v1.26.0 (Love et al. 2014).

## Data access

All raw and processed sequencing data generated in this study have been submitted to the NCBI Gene Expression Omnibus (GEO; https://www.ncbi.nlm.nih.gov/geo/) under accession number GSE229157. Scripts used in the generation and analysis of data for this project can be found at GitHub (https://github.com/jonathan-wells/metazoan-znfs; https://github.com/jonathan-wells/finz-znf) and as Supplemental Code.

## Supplementary Material

Supplement 1

Supplement 2

Supplement 3

Supplement 4

Supplement 5

Supplement 6

Supplement 7

Supplement 8

Supplement 9
